# The Predictive Value of the AQ and the SRS-A in the Diagnosis of ASD in Adults in Clinical Practice

**DOI:** 10.1007/s10803-020-04699-7

**Published:** 2020-10-01

**Authors:** M. L. Bezemer, E. M. A. Blijd-Hoogewys, M. Meek-Heekelaar

**Affiliations:** 1 INTER-PSY, Verlengde Meeuwerderweg 7, 9723 ZM Groningen, The Netherlands; 2 Department of Developmental Psychology, University of Groningen, Grote Kruisstraat 2/1, 9712 TS Groningen, The Netherlands; 3 Present Address: Private Practice PP-Noord and Psychology Academy Groningen, Vechtstraat 62, 9725 CW Groningen, The Netherlands

**Keywords:** ASD, AQ, SRS-A, Predictive value

## Abstract

Questionnaires are widely used in autism assessment. However, their psychometric properties are generally not evaluated in clinical practice, and the comparability and applicability of such research is limited because questionnaires are often not simultaneously evaluated. This certainly pertains to predictive values which are highly population and setting specific. This study evaluated the power of AQ and SRS-A in predicting an ASD diagnosis within the same clinical population. The patient records of 92 adults, referred for autism assessment, were analyzed. The AQ proved somewhat better than the SRS-A at discriminating and predicting autism. The predictive values of both questionnaires were lower than reported in general population studies. Psychometric results in core publications appear less representative for clinical practice.

## Introduction

Epidemiological research shows that autism spectrum disorder (ASD) occurs in approximately 1% of the adult population (Brugha et al. 2011). For children, this figure has recently been adjusted upward and ranges from 1.68% (Baio et al. 2018) to 2.47% (Xu et al. 2018), which is partly due to differing autism survey methodologies (Fombonne 2018). The diagnostics of ASD take place in specialized departments, as well as in more general departments within the mental healthcare system. We suspect that ASD cases are more frequently missed in the more general departments, as clinicians in these facilities are, on average, less sensitive to recognizing ASD. Explanations include the often wider diversity of reasons for referring to such departments, the likelihood that ASD is concealed by co-morbid psychopathology (diagnostic overshadowing), and greater subtlety in the manifestations of ASD. Above-average intelligence, an optimal environment, and acquired camouflaging behaviors (masking, compensation, and assimilation; Hull et al. 2017) also make it difficult to recognize the symptoms of ASD (Bargiela et al. 2016). When ASD remains undetected, it regularly leads to secondary problems (e.g., depression, anxiety, and burn-out), reduced quality of life (Bargiela et al. 2016), and increased financial costs (Horlin et al. 2014).

Following (inter) national guidelines, the diagnostics of ASD requires a comprehensive assessment (Kan et al. 2013; NICE 2012). For adults without cognitive disabilities, this consists of a general psychiatric examination of the characteristics of ASD and possible co-morbidity. Also, a thorough (early) developmental history is assessed with a relative (preferably the primary caregiver) and possibly an interview with another informant (e.g. a partner or friend). Additionally, questionnaires may be used. Questionnaires can play a role in decisions concerning whether a referral for ASD assessment is needed (i.e., for screening purposes), as well as in support of formal ASD assessment (i.e., for diagnostic purposes). With regard to the predictive power of questionnaires concerning the ultimate diagnosis, the concepts of sensitivity, specificity, positive predictive value (PPV), and negative predictive value (NPV) play an important role (Parikh et al. 2008).

Sensitivity refers to the percentage of positive test results among the people with the condition. Specificity refers to the percentage of negative test results among people without the condition. From a different perspective, PPV refers to the percentage of people with the condition among the positive test results, and NPV refers to the percentage of people without the condition among the negative test results. Patients are thus taken as the source for calculating sensitivity and specificity, while test results are taken as the source for calculating PPV and NPV. It is interesting to note that, while sensitivity and specificity are often mentioned in research on the predictive value of questionnaires, PPV and NPV are much less commonly mentioned. The latter two properties nevertheless seem quite relevant to clinical practice, especially when it comes to using questionnaires as part of screening or diagnosing. After all, when a questionnaire is used for such purposes it is primarily important to know how much value can be assigned to the results of the questionnaire (given that the diagnosis is not yet known). If an individual scores above the cut-off point on a questionnaire, it is important to know the accuracy of the estimate that the individual actually does have the condition (PPV) or that the result is a false alarm (false positive = 1 − PPV). Conversely, if an individual scores below the cut-off point on a questionnaire, it is important to know the accuracy of the estimate that the individual actually does not have the condition (NPV) or that the result is a missed patient (false negative = 1 − NPV). Ideally, all of these predictive values of a questionnaire should be as high as possible. In practice, however, high sensitivity often comes at the expense of specificity, and vice versa. This also applies for PPV and NPV. Depending on the specific use of the questionnaire, a choice must be made concerning which values are to be assigned the most weight.

Various ASD questionnaires for adults are used in clinical practice, including the Autism-spectrum Quotient (AQ; Baron-Cohen et al. 2001), the Ritvo Autism Asperger Diagnostic Scale-Revised (RAADS-R; Ritvo et al. 2011), the Social Responsiveness Scale Adult version (SRS-A; Constantino and Todd 2005), and the Social Communication Questionnaire (SCQ; Rutter et al. 2003). Of these instruments, the AQ and the SRS-A appear to be the most commonly used in the Netherlands. These questionnaires differ in several aspects, such as the length of the questionnaire (50 or fewer items for the AQ, as compared to 64 items for the SRS-A), the cost of the questionnaire (open source for the AQ, as opposed to paid for the SRS-A), the availability of standards (only a cut-off score for the AQ, whereas there are standardized scores available for the SRS-A) and the psychometric properties of these questionnaires, including the predictive values. Looking at the AQ, this questionnaire possesses satisfactory to good psychometric qualities (Baron-Cohen et al. 2001; Hoekstra et al. 2008). The sensitivity of the AQ is .79, and the specificity is .98 (with a cut-off value of 32; Baron-Cohen et al. 2001). When PPV and NPV are calculated with MedCalc (MedCalc Software, Belgium) for aforementioned studies, both values are .93. The SRS-A is the adult version of the SRS. The SRS has proven itself as a reliable questionnaire with good psychometric qualities when applied for detecting possible ASD in children (Bölte et al. 2008, 2011). According to the Dutch professional manual, the adult version also has satisfactory psychometric qualities (Noens et al. 2012). The sensitivity of the SRS-A is .85, and the specificity is .82 (with a cut-off value of 54). When PPV and NPV are calculated with MedCalc, they are .20 and .99, respectively.

The above-mentioned psychometric data are based on the relevant core publication or professional manual. Diverging results have nevertheless been reported in other studies. As illustrated by different researchers (e.g. Sizoo et al. 2015), it should be noted that predictive values are strongly dependent on the research situation in which the questionnaires are investigated. We would like to highlight three main factors that have a tremendous effect on predictive values of questionnaires: (1) the nature of the ASD and non-ASD groups that are being compared, (2) the rate of ASD in the total research group, and (3) the used cut-off point of the questionnaire at stake.

With regard to the first point, a questionnaire will have better predictive values when there is a greater difference in the severity of ASD between the two groups compared. Most core publications or professional manuals take patients with ASD in the strictest sense and compare them to a non-ASD comparison group without any other diagnoses (e.g. Baron-Cohen et al. 2001; Noens et al. 2012; Wakabayashi et al. 2006). This is less representative of clinical practice, however, where there is a greater need to differentiate between patients from the broader autism spectrum and those with other psychiatric disorders whose symptoms can overlap with autism. With regard to the second point, it should be noted that the rate of ASD in the study (i.e. the percentage of people with ASD in the total research group in that specific study) has an influence on PPV and NPV. Assuming that all other factors are constant, PPV increases with higher ASD rate, while NPV decreases (Parikh et al. 2008). In most core publications or professional manuals, ASD rates appear to be relatively low, as these publications draw upon a large group of a non-ASD comparison group without any other diagnoses relative to a small group of patients with ASD (f.i. in the SRS-A manual the ASD rate was 5%; the PPV is low, at .20). These ASD rates are lower than what is usually observed within the general mental healthcare system. If the questionnaire is used in specialized ASD departments, where all patients are suspected of ASD, the ultimate rate of ASD will be even higher (e.g. Ashwood et al. 2016; Woodbury-Smith et al. 2005). With regard to the third point, a higher cut-off score (indicative for more problems) generally leads to a lower sensitivity, a higher specificity, less false alarms and more missed patients (Wassertheil-Smoller and Smoller 2015).

Results concerning predictive values of the AQ and the SRS-A do indeed vary considerably in different groups, with different ASD rates, and with different cut-off scores (see Table 1, with both core publications marked in italic). Concerning the composition of groups, some studies included ASD samples versus the general population (which is common in research settings), other studies included clinical samples suspected for ASD resulting in a subgroup with and a subgroup without ASD (which is common in clinical settings). As a result, the amount of ASD characteristics, operationalized by the mean AQ or SRS-A score, differed both between and within studies. For example, looking at the AQ between studies, the minimum mean score is 16.40 (for a general population group; Baron-Cohen et al. 2001) and the maximum mean score is 37.90 (for an ASD group; Wakabayashi et al. 2006). Within studies, the minimum mean difference score is 2.45 (Conner et al. 2019) and the maximum mean difference score is 19.4 (Baron-Cohen et al. 2001; Wakabayashi et al. 2006). The smallest differences between research groups are seen in clinical settings. With regard to ASD rates, different settings (research versus clinical) resulted in huge differences (ranging from 5 to 73%). The former two factors—group composition and ASD rate—also lead to different optimal cut-off points. Most studies calculated an optimal cut-off point, while some only used the typical cut-off point as published in the core publication or professional manual (Ashwood et al. 2016; South et al. 2017). Complicating matters even more, all three factors—group composition, ASD rate and cut-off point—can vary independently over the different studies. As a result, most studies cannot truly be compared to one another.

**Table 1:** Summary of predictive values of AQ and SRS-A as self-report measure in different studies with adults

Study	Population characteristics	M (SD) Questionnaire	Cut-off (raw score)	AUC^a^	Sensitivity	Specificity	PPV | FP	NPV | FN	ACC
AQ-50									
*Baron-Cohen et al. *(2001)	*Research setting, adults already diagnosed with ASD (HFA or AS, n = 58) vs general population (n = 174)* *ASD rate = 25%*	*ASD: 35.80 (6.50)* *GP: 16.40 (6.30)* *diff score = 19.40*	*32*	*NR*	*.79*	*.98*	*93% | 7%*^*b*^	*93% | 7%*^*b*^	*93%*^*b*^
Kurita et al. (2005)	Research setting, mild ASD (HPDD, n = 25) vs general population (n = 215) ASD rate = 10%	ASD: 29.60 (6.30) GP: 22.20 (6.60) diff score = 7.40	26	NR	.76	.71	24% | 76%	96% | 4%	72%^b^
Woodbury-Smith et al. (2005)	Clinical setting, ASD referrals: ASD (HFA or AS, n = 73) vs non-ASD (received other psychiatric diagnosis, n = 27) ASD rate = 73%	ASD: 35.62 (6.63) non-ASD: 26.22 (9.39) diff score = 9.40	26	.78	.95	.52	84% | 16%	78% | 22%	83%^b^
Wakabayashi et al. (2006)	Research setting ASD (HFA or AS, n = 57) vs general population (n = 194) ASD rate = 23%	ASD: 37.90 (5.31) GP: 18.50 (16.31) diff score = 19.40	33	NR	.88	.97	90% | 10%^b^	96% | 4%^b^	95%^b^
Ashwood et al. (2016)	Clinical setting, ASD referrals: ASD (n = 346) vs non-ASD (received other psychiatric diagnosis, n = 130) ASD rate = 73%	ASD: 34.90 (8.20) non-ASD: NR diff score = NA	26	.56^c^	.88	.20	76% | 24%	36% | 64%	69%^b^
			32	.56^c^	.71	.35	76% | 24%	29% | 71%	61%^b^
Conner et al. (2019)	Clinical setting, ASD referrals: ASD (n = 31) vs non-ASD (received other psychiatric diagnosis, n = 62) ASD rate = 33%	ASD: 30.65 (5.89) non-ASD: 33.10 (5.33) diff score = 2.45	32	.40	.55	.34	29% | 71%^b^	61% | 39%^b^	41%^b^
			33.5	NR	.45	.52	32% | 68%^b^	66% | 34%^b^	49%^b^
SRS-A									
*Noens et al. *(2012) *(Dutch manual)*	*Research setting, adults already diagnosed with ASD (n = 74) vs general population (n = 1449)* *ASD rate = 5%*	*ASD: 93.10 (32.10)*^*d*^ *GP: 36.74 (22.66)* *diff. score = 56.36*	*54*	*.91*	*.85*	*.82*	*20% | 80%*^*b*^	*99% | 1%*^*b*^	*82%*^*b*^
Mandell et al. (2012)	Clinical setting, hospitalized patients with schizophrenia (n = 127) vs hospitalized patients with schizophrenia + ASD (n = 14) ASD rate = 10%	ASD: 100.20 (32.70) Non-ASD: 76.50 (32.50) diff score = 23.70	84	.72	.86	.60	19% | 81%	97% | 3%	84%^b^
South et al. (2017)	Research setting, university students already diagnosed with ASD (n = 40) vs high anxiety (n = 56) and controls (no psychiatric history, n = 29) ASD rate = 32% (ASD vs ANX + CON) or 42% (ASD vs ANX)	ASD: 84.60 (28.83) Anxiety: 67.73 (24.19) Controls: 41.28 (18.85) diff score (ASD-ANX) = 16.87 or diff score (ASD-CON) = 43.32	NR	NR	.75	.66 (ASD vs ANX + CON)	51% | 49%^b^	85% | 15% ^b^	69%^b^
					NR	.48 (ASD vs ANX)	51% | 49%^b^	73% | 27%^b^	NA

Sizoo et al. (2015), study (clinical setting, adults suspected of ASD, ASD rate = 66%) not included here, since it does not concern AQ-50, but AQ-10 and AQ-28. Conner et al. (2019) had a non-ASD groups that scored higher than the ASD group on the AQ: this finding is remarkably different from all other studies reported here

*ACC* accuracy (correct classification), *ANX* anxiety, *AS* Asperger syndrome, *ASD* autism spectrum disorder, *CON* control, *diff score* difference in mean score, *FN* false negative, *FP* false positive, *GP* general population, *HFA* high-functioning, *HPDD* high-functioning pervasive developmental disorder, *NA* not applicable, *NR* not reported

^a^At optimum cut-off

^b^Not reported in the study, but calculated by the current authors, on basis of the reported results in the article

^c^Not reported in the study whether the AUC is for AQ cut-off 26 or 32

^d^Not reported for the total ASD group (n = 74), but for a smaller group (n = 60)

This implies that the predictive values of questionnaires (as known from core publications or professional manuals) are not necessarily representative for clinical practice. In many cases, the values are based primarily on the general population for screening purposes. For diagnostic purposes, however, it is important to evaluate instruments in clinically suspected populations. It is also important to examine the value of the instrument in patients who are still in the assessment phase, and not already diagnosed with ASD (Bishop and Seltzer 2012; Sizoo et al. 2015), considering that patients who already know that they have ASD might complete questionnaires differently than would patients who do not yet know that they have ASD. The latter group comprises the very group of patients to whom these questionnaires will be presented in clinical practice and for whom the predictive value is relevant. It should be noted that, in the professional manuals and core publications for the AQ and SRS-A, patients were already aware of their ASD diagnoses at the time that they completed the questionnaire.

To summarize, the predictive values of different questionnaires—in this case, their ability to predict a clinical ASD diagnosis—are difficult to compare over studies, since different settings and research groups are involved, as was illustrated in Table 1. In order to properly compare two questionnaires, they should be analyzed in the same setting and group. The present study is the first to compare the AQ and the SRS-A within the same clinical setting. There has been, however, one study within a non-clinical population of students, in which both were compared as screening instruments for the broader autism phenotype (people with ASD traits without a true ASD diagnosis, and thus with a very mild severity). In that study, the SRS-A had the strongest criterion validity (Ingersoll et al. 2011). The goal of the present study is to compare the value of both ASD questionnaires in correctly predicting the ASD diagnosis in a clinically suspected adult population. This may help in determining which questionnaire to use in the assessment phase for adults in mental healthcare.

## Methods

### Participants

The research population consisted of adult patients who were assessed for suspected ASD (N = 147), in the period from April 2015 through April 2016, at INTER-PSY, a facility for outpatient general mental healthcare. Patients were referred by a medical professional (e.g. GP, psychologist or psychiatrist). All individuals with clinical referrals were considered for participation. Exclusion criteria were as follows: incomplete research data (n = 25), non-compliance to the ASD assessment protocol (n = 23), diagnostic drop-out (n = 3), the presence of an acute mental health condition (e.g. manic or psychotic states; n = 0) or evidence of low intellectual functioning (n = 4 with intellectual disability established, TIQ ≤ 70). With regard to the last criterion, former intelligence testing results were taken into account or the exclusion criterion was judged based on clinical impression and the highest level of education completed. In doubtful cases, intelligence testing was performed with the WAIS-IV-NL (Wechsler 2012; n = 23). The research group ultimately consisted of 92 patients (18–62 years), of which 63 received an ASD diagnosis (68%), including 19 patients (30%) with a co-morbid psychiatric disorder (see Table 2, also for the diagnoses of patients who did not receive an ASD diagnosis). Most of the ASD diagnoses were at the DSM-5 severity level of 1, which corresponds to the lowest level of support needed. Note that there is no overall calibration for this metric; it merely reflects the clinician's estimate of overall functioning.

**Table 2:** Characteristics of ASD group versus non-ASD group

	ASD (*n* = 63)	Non-ASD (*n* = 29)
Sex: male	36 (57%)	17 (58%)
Age: *M* (*SD*)	33.68 (12.40)	33.14 (12.30)
ASD severity level according to DSM-5		
Social communication	Level 1: 86% Level 2: 12% Level 3: 2%	
Restricted, repetitive behaviors	Level 1: 79% Level 2: 18% Level 3: 3%	
Other psychiatric diagnosis^a^		
Depressive disorder	11	10
Anxiety disorder	6	6
ADHD	2	6
Personality disorder	3	3
Substance-related & addictive disorder	1	2
Dissociative disorder	0	1
No psychiatric disorder	0	1

There was no statistical significant difference between the two research groups with regard to sex ratio or age

^a^In some cases participants had more than one diagnosis

### Procedure

Before the start of the study, permission was granted by the local hospital’s medical ethics committee. All participants underwent an extensive diagnostic assessment, using multiple measures and multiple informants, focusing on ASD and possible other psychiatric disorders. The DSM-5 criteria for ASD were used (APA 2013), and the Dutch national ASD guideline was followed (Kan et al. 2013). The latter was developed in close collaboration with the National Institute for Health and Care Excellence (NICE). The diagnostic assessment was performed by a team of independent, experienced clinicians (n = 29). Their diagnostic conclusion was based on a thorough developmental history and the use of a Dutch ASD diagnostic interview, namely the NIDA (Vuijk 2014) or the ASD DSM-5 interview (Spek 2015). In both semi-structured instruments, the ASD symptoms are systematically assessed. For each DSM criterion, main questions and correspondent key examples are asked in order to evaluate the presence or absence of that criterion in present and childhood. Besides that, clinical impressions of the clinician are taken into account.

At the start of the assessment phase, patients completed a number of questionnaires, including the AQ and the SRS-A (care-as-usual). These questionnaires were processed by independent collaborators (psychologists). For research purposes, a minimal intervention was made in this care-as-usual process. Before receiving the results of the questionnaires and before sharing the diagnosis with the patient concerned, the clinician shared his/her diagnostic conclusion with the researcher. At that time, this conclusion was based on the elaborate clinical interviews used. The questionnaire results did not contribute to this diagnostic conclusion, preventing confounding of the research results. After sharing their diagnostic conclusion with the researcher, the clinician received the questionnaire results. When the diagnosis was given to the patient, the results of the questionnaires were also incorporated. A later check showed that no diagnostic decisions were changed by the clinicians after receiving the questionnaire results.

### Instruments

The Autism-spectrum Quotient (AQ; Baron-Cohen et al. 2001; Hoekstra et al. 2008) is a self-report questionnaire for adults that measures symptoms that are indicative of ASD. The questionnaire examines several dimensions: Social skill, Attention switching, Attention to detail, Communication, and Imagination. The AQ consists of 50 questions. The respondent determines the extent to which a symptom is personally applicable on a 4-point Likert-scale: ‘definitely agree’, ‘slightly agree’, ‘slightly disagree’, and ‘definitely disagree’. All answers are dichotomized for processing. More specifically, they are transformed to 0 (not indicative of ASD) or 1 (indicative of ASD). This produces a scoring range from 0 to 50, with higher scores indicating more ASD characteristics. In the Dutch multi-disciplinary guideline for ASD in adults (Kan et al. 2013), a cut-off point of 32 is recommended as indicative of ASD for purposes of screening in the general population (see also Baron-Cohen et al. 2001), and a cut-off point of 26 for clinical practice (see also Woodbury-Smith et al. 2005). As stated in the introduction, the AQ possesses satisfactory psychometric qualities (Baron-Cohen et al. 2001; Hoekstra et al. 2008).

The Social Responsiveness Scale for Adults (SRS-A; Constantino and Todd 2005) is a self-report questionnaire for adults that measures the degree of social responsiveness. Several dimensions that are characteristic of ASD are examined: Social awareness, Social communication, Social motivation, and Restricted interests and repetitive behavior. The Dutch version of the SRS-A consists of 64 questions (in contrast to the English version, which consists of 65 items). Each question is scored on a 4-point Likert-scale: ‘not true’, ‘sometimes true’, ‘often true’, and ‘almost always true’. The raw total scores range from 0 to 192. In the Dutch professional manual (Noens et al. 2012), a cut-off point of 54 is recommended as indicative of ASD for purposes of screening in the general population. No cut-off point is known for clinical practice. As mentioned in the manual, a higher cut-off point can be chosen if desired in clinical settings, in order to limit the over-identification of ASD (i.e., false alarms), given the greater ASD rates in clinical practice. Our study uses raw scores, to facilitate comparability of the psychometric data. As stated in the introduction, the SRS-A possesses satisfactory psychometric qualities (Noens et al. 2012).

### Data analysis

After receiving the ultimate diagnosis, the participants were divided into two research groups: patients with ASD (the ASD group) and patients without ASD (the non-ASD group). Independent-samples t-tests were conducted to determine whether the results of the ASD questionnaires (in terms of total scores and scale scores) differed between these groups. Pearson’s correlations were calculated as an indicator of convergent validity (i.e., the extent to which the total scores and scale scores of AQ and SRS-A were correlated). As an indicator of internal consistency, Cronbach’s alpha for total scores and scale scores of AQ and SRS-A were calculated. To examine the predictive value of both questionnaires for obtaining an ASD diagnosis, receiver operating characteristics (ROC) curves were calculated for the total scores. A ROC curve indicates the sensitivity and specificity associated with every possible cut-off value for a questionnaire. The area under the curve (AUC) is a measure of predictive validity that can be used to compare the predictive value of different questionnaires. The result can range between 0 and 1, with an AUC value of .50 representing a predictive value at chance level. The Youden index was also calculated for both questionnaires (Youden 1950). This index provides the cut-off point for the highest possible sensitivity in combination with the highest possible specificity (Krzanowski and Hand 2009). The positive predictive value (PPV), the negative predictive value (NPV) and their respective accuracy (ACC) associated with this ideal cut-off point were subsequently calculated for both questionnaires. Crosstabs for both questionnaires were calculated and analyzed (Chi-square test). ROC analyses were performed in MedCalc for Windows, Version 18.5 (MedCalc Software, Ostend, Belgium), and SPSS 26.0 was used for all other analyses. All statistical tests were two-tailed with an alpha of .05, and where appropriate corrected for multiple testing using Bonferroni (alpha divided by number of tests). Where applicable, effect sizes (Cohen’s d) were calculated (Cohen 1988).

## Results

### AQ and SRS-A Compared in the ASD and Non-ASD Group

The average total scores and scale scores of the AQ and SRS-A for both research groups are presented in Table 3. All average total scores and scale scores for the ASD group were higher than for the non-ASD group, indicating more ASD characteristics. After correcting for multiple testing, the AQ total score and two AQ scales (Social skill and Communication) were statistically significantly higher for the ASD group, with a large effect size. For the SRS-A, this was only the case for one scale (Social motivation), with a medium effect size. The association between the two questionnaires was high, with a correlation of .79 for the total scores (*p* < .001). The inter-scale correlations between the AQ scales and SRS-A scales ranged from moderate to high (p < .001), except for three low correlations (p < .05) (see Table 4). The intra-scale correlations of the AQ are lower than for the SRS-A. The Cronbach’s alpha for the AQ total was .87 and for the SRS-A .93. The Cronbach’s alpha for the AQ scales were for Social skills .75, Attention switching .76, Attention to detail .75, Communication .65, and Imagination .55. The Cronbach’s alpha for the SRS-A scales were for Social awareness .80, Social communication .82, Social motivation .81, and Restricted interests & repetitive behavior .81.

**Table 3:** AQ and SRS-A scores for ASD group versus non-ASD group

	ASD (*n* = 63) *M* (*SD*)	Non-ASD (*n* = 29) *M* (*SD*)	Effect size (Cohen’s *d*)
AQ total**	29.17 (7.75)	20.97 (8.13)	1.04
AQ scales			
Social skill**	6.70 (2.19)	3.86 (2.72)	1.20
Attention switching	7.10 (2.43)	5.66 (2.79)	.57
Attention to detail	5.48 (2.62)	4.14 (2.34)	.53
Communication**	5.38 (2.22)	3.45 (2.16)	.88
Imagination	4.52 (2.14)	3.86 (2.15)	.31
SRS-A total	83.92 (24.25) *70*.*87* (*10*.*76*)	67.42 (28.04) *63*.*59* (*12*.*56*)	.65 .*64*
SRS-A scales			
Social awareness	22.30 (7.83) *66*.*44* (*11*.*68*)	18.14 (8.37) *60*.*59* (*12*.*80*)	.52 .*49*
Social communication	29.08 (8.36) *70*.*56* (*9*.*93*)	23.48 (10.60) *63*.*83* (*12*.*36*)	.61 *.63*
Social motivation*	18.83 (5.89) *70*.*17* (*11*.*20*)	14.62 (6.64) *62*.*59* (*12*.*26*)	.69 .*66*
Restricted interests & repetitive behavior	13.71 (6.16) *68*.*78* (*13*.*85*)	11.17 (6.66) *63*.*24* (*14*.*71*)	.40 .*39*

^*^p = .004, **p < .001, corrected for multiple testing, using Bonferroni correction *p* < *α* = .05/11

For clinical interpretation, not only raw scores but also T-scores (*in italic*) are presented for the SRS-A. The conclusions concerning statistical significant differences are the same

**Table 4:** Inter- and intra-scale correlations of AQ and SRS-A

	SRS-A					AQ					
	Total	Social awareness	Social communication	Social motivation	Restricted interests & repetitive behavior	Total	Social skill	Attention switching	Attention to detail	Communication	Imagination
AQ											
Total	.79	.72	.76	.64	.62		.78	.74	.58	.77	.63
Social skill	.62	.48	.59	.68	.40			.51	.22^a^	.60	.36
Attention switching	.67	.58	.61	.55	.58				.31^a^	.41	.30^a^
Attention to detail	.34	.35	.25^a^	.22^a^	.35					.25^a^	.20^b^
Communication	.67	.64	.65	.50	.51						.44
Imagination	.48	.47	.56	.24^a^	.31						
SRS-A											
Total		.90	.94	.77	.85						
Social awareness			.81	.52	.72						
Social communication				.66	.71						
Social motivation					.53						
Restricted interests & repetitive behavior											

All correlations are statistically significant at *p* < .001, except for ^a^, which are statistically significant at *p* < .05, and ^b^, which is a trend *p* < .06

### Predictive Values of the AQ and SRS-A

**Fig. 1:**
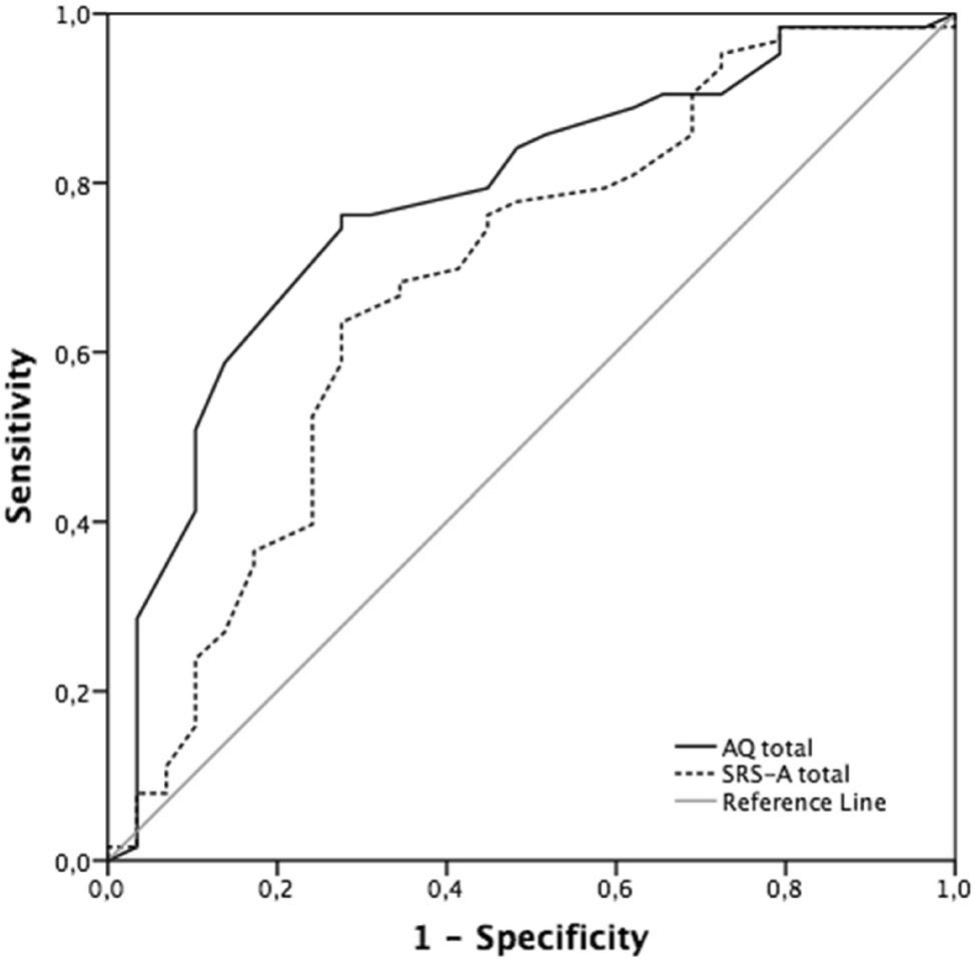
ROC curve of AQ and SRS-A

ROC curves of AQ and SRS-A total score are illustrated in Fig. 1. Analyses revealed an AUC of .78 (acceptable discrimination) for the AQ and an AUC of .69 (poor discrimination) for the SRS-A (Hosmer et al. 2013). Both differed statistically significantly from chance level (*p* = .001 and *p* = .003, respectively). The AUC of the AQ was statistically significantly higher than that of the SRS-A (*p* = .017). Using the Youden index, an optimal cut-off point of 26 was found for the AQ, and an optimal cut-off point of 81 for the SRS-A. The various predictive values of both questionnaires for these cut-off points are presented in Table 5. The table also presents predictive values for the cut-off points, as proposed in the core publication or professional manual (for the AQ: 32, and for the SRS-A: 54).

**Table 5:** Predictive values of different AQ and SRS-A cut-off scores

	Cut-off	AUC	Sensitivity	Specificity	PPV | FP	NPV | FN	ACC
AQ	**26**	.**778**	.**76**	.**72**	**86% | 14%**	**58% | 42%**	**75%**
	32		.41	.90	90% | 10%	41% | 59%	
SRS-A	54		.89	.31	74% | 26%	56% | 44%	
	**81**	.**684**	.**63**	.**72**	**83% | 17%**	**48% | 52%**	**66%**

The optimal cut-off point (indicated in boldface) is the score that maximizes the sum of sensitivity and specificity

*AUC* area under the curve, *FN* false negative, *FP* false positive, *PPV* positive predictive value (correctly classified patients with ASD), *NPV* negative predictive value (correctly classified patients without ASD)

There was a statistically significant association for both AQ and SRS-A between test result and ultimate diagnosis (ASD or non-ASD) (AQ: χ^2^ (1) = 19.70, *p* < .001; SRS-A: χ^2^ (1) = 10.26, *p* = .001) (see Table 6). So, both predicted an ASD diagnosis better than chance, with a medium strength of association (AQ: φ = .46; SRS-A: φ = .33). Looking at individual cases, in 16 out of 92 (17%), the AQ and SRS-A test result led to different classifications (ASD versus non-ASD). In 12 of these 16 cases the AQ predicted the ultimate diagnosis correctly, in 4 cases the SRS-A did so.

**Table 6:** Crosstab test result versus diagnosis

	ASD diagnosis	Non-ASD diagnosis
AQ indicative result (≥ 26)	48	8
AQ non-indicative result (< 26)	15	21
SRS-A indicative result (≥ 81)	40	8
SRS-A non-indicative result (< 81)	23	21

## Discussion

The aim of this study was to examine the predictive value of two commonly used ASD self-report questionnaires—the AQ and the SRS-A—in the assessment phase in clinical practice. In other words, we examined their ability to predict a clinical ASD diagnosis. After all, data from more general psychometric research using carefully screened comparison groups are not necessarily representative for a clinical setting. The study was conducted among 92 adult patients who had been referred for ASD assessment to a general facility for outpatient mental healthcare. During the assessment phase, they also completed an AQ and an SRS-A. At that time, their ultimate diagnoses were not yet known. After the assessment phase, 68% were identified as having ASD.

### Implications

The AQ and SRS-A total scores were highly correlated (*r* = .79). As such, they can be assumed to measure a similar overall construct (they display good convergent validity). Previously, this was only demonstrated for the child versions of these questionnaires (*r* = .64; Armstrong and Iarocci 2013). The inter-scale correlations between AQ and SRS-A suggest large positive associations, except for the AQ scales Attention to detail and Imagination. Thus, in contrast to the total scores, the specific scales of the AQ and SRS-A seem to represent somewhat different underlying constructs. Indeed, looking at the scale descriptions, the AQ is unique in measuring attention for details and imagination, while the SRS-A is so in measuring social motivation. Both measure social skills and communication, and rigidity. The intra-scale correlations are acceptable for both questionnaires. While the AQ had some low correlations, suggesting measuring perhaps a non-related construct (Attention to detail); the SRS-A had some high correlations, suggesting measuring not distinct enough constructs (Social communication and Social awareness) (Aaronson et al. 1993). The internal consistency of the SRS-A is excellent for the total score and good for the scale scores. The internal consistency of the AQ is good for the total score and acceptable for the scale scores, except for Communication (questionable) and Imagination (poor).

The scores of the ASD group were higher than those of the non-ASD group on both questionnaires. However, the majority of the scales did not show a statistically significant difference between ASD and non-ASD. This is not surprising, since our research group consisted of people whom were all suspected for ASD. In such a group, smaller differences can be expected than, for instance, when comparing ASD to the general population. Although carefully designed research studies comparing ASD groups to comparison groups or groups from the general population suggest strong differences in these constructs, these differences are much weaker in clinical practice. This supports dimensional models of ASD symptoms. While ASD is conceptualized in the DSM-5 (APA 2013) as a categorical construct with clear delineation between affected and unaffected individuals, there is growing evidence that ASD is best conceptualized as being dimensional—with ASD traits being continuously distributed in the population—rather than categorical (Kim et al. 2019). However, categorical and dimensional classification of autism need not be mutually exclusive; they can be complementary, as they may explain different aspects of the condition (Abu-Akel et al. 2019). This is by no means the last word on this matter, since the method of classification used has consequences for evaluating questionnaire results. Should one consider cut-off points, dimensional scores (such as percentile scores) or a combination of both?

Both questionnaires predicted the ultimate ASD diagnosis better than chance. But, the AQ proved to be somewhat better at discriminating and predicting the ASD diagnosis than the SRS-A in this study. Not only the total AQ score, but also two scale scores (Social skill and Communication) were statistically significantly different between the ASD group and the non-ASD group. In contrast, the SRS-A revealed a statistically significant difference for only one scale (Social motivation). Moreover, the corresponding effect sizes for the AQ were large, while the one for the SRS-A was medium. In 83% of the individual cases, there was agreement between both instruments concerning the classification (ASD or non-ASD). Where they differed, the AQ correctly predicted the ultimate diagnosis more frequently (12 out of 16 cases). The predictive value of the AQ (based on the AUC) was statistically significantly larger than that of the SRS-A. The predictive value of the SRS-A can be considered as ‘unsatisfactory’ (Hosmer et al. 2013). For the optimal cut-off point, the PPV showed that the AQ correctly classified 86% of the patients as having ASD. The SRS-A yielded a comparable 83%. Based on the NPV, the AQ correctly classified ‘only’ 58% of the patients as not having ASD. For the SRS-A, this figure was 48%, which falls below chance level. The latter results highlight the fact that questionnaires cannot replace diagnostic assessment by trained clinicians. Based on the aforementioned PPV and NPV, we conclude that instances of ‘false alarms’ (false positives) and particularly ‘missed patients’ (false negatives) are still quite common. False alarms (total scores above the cut-off point, in the absence of an ASD diagnosis) occurred in 14% of all cases for the AQ and in 17% of all cases for the SRS-A. The percentage of missed patients (total scores below the cut-off point, in the presence of an ASD diagnosis) was much higher. For the AQ, this was 42% and, for the SRS-A, 52% (more than half). Both questionnaires thus appear better suited to predict an ASD diagnosis than they are to rule out such a diagnosis. The fact that the rate of missed patients was so high is remarkable. This is believed to be a consequence of a clinical setting, where the prevalence rate of ASD is much higher than in a research setting. In the latter case, most commonly a small group of people with ASD is compared to a large group from the general population. The chance of missing patients in such a setting is obviously lower than in a setting with a much higher rate of ASD. This trend of a lower prevalence rate leading to a lower rate of missed patients can be observed in the results as presented in Table 1, with one exception (Woodbury-Smith et al. 2005). However, note that other factors, such as the difference in ASD characteristics between the compared groups (see “Introduction” section), have a combined effect on the predictive values.

It is difficult to provide a general recommendation to clinicians for which questionnaire to use. Both questionnaires have their own strengths. The SRS-A had better reliability—a higher internal consistency was found—and offers norm-referenced scores. The AQ had better criterion and predictive validity: it was better in differentiating ASD from non-ASD and better in predicting the ultimate diagnosis. The choice of which questionnaire to prefer also depends on which information is desired by the clinician: the scales of both questionnaires cover somewhat different areas. For instance, the SRS-A seems more linked to DSM criteria and explicitly covers social motivation, while the AQ also gives explicit attention to other criteria seen in clinical practice, such as certain information processing characteristics. But, as explained in the introduction, results concerning psychometric data can differ widely by setting. So, deciding which questionnaire is most preferable also depends on the setting in which it will be used. Unfortunately, such information is mostly lacking. This calls for more research. Ideally, the AQ and SRS-A should be compared simultaneously, within the same population, in many more different settings. Only then could one enhance the understanding of the ‘practical’ psychometrics of these ubiquitous questionnaires. Only then better-informed choices could be made in which instrument to use in one’s specific setting. Regardless of the choice which questionnaire to use, one also has to make a decision on which cut-off point to use. For example, in our study, the optimal cut-off point for the SRS-A was much higher than the cut-off point suggested in the professional manual for screening purposes in the general population (81 vs 54). As suggested in the professional manual, a higher cut-off point can limit the over-identification of ASD (i.e., false alarms). Indeed, the optimal higher cut-off point for the SRS-A found in our study resulted in fewer false alarms (17% vs 26%), although it also resulted in more missed patients (52% vs 44%). For the AQ, former research already calculated an optimal cut-off point for use in clinical practice (i.e., 26); this is the same as we have found in our research. In contrast to the SRS-A, this optimal cut-off point for the AQ in clinical practice is lower than the one suggested for the general population. This has a reverse effect, namely more false alarms (14% vs 10%) and fewer missed patients (42% vs 59%). Clinicians should decide which is most preferable within their practice: identifying patients (true positives) but having more false alarms (false positives) or ruling out the condition (true negatives) but having more missed patients (false negatives). Depending on this decision, one could use different cut-off points.

As mentioned earlier, the predictive values of questionnaires are influenced by differences in ASD severity and ASD rate in the groups of interest. In clinical practice, when ASD is already suspected, there is a relatively smaller difference in ASD severity combined with a higher ASD rate in the group at stake. The results of our study are relevant for such a situation. However, if the goal is to screen for ASD in a general population and determine whether ASD assessment is needed at all (using the questionnaire as a gateway), it is better to consider psychometric data from professional manuals and core publications (e.g., Baron-Cohen et al. 2001; Noens et al. 2012). These involve a relatively larger difference in ASD severity combined with a relatively lower ASD rate in the group at stake. In screening situations, it is also important to consider whether greater value should be assigned to PPV (related to false alarms) or to NPV (related to missed patients), with regard to the social or financial costs arising when individuals are referred for ASD assessment (weighed against the negative consequences of missing a patient).

### Strengths and Limitations

To the best of our knowledge, this is the first study to make a direct comparison of AQ and SRS-A within the same clinically ASD suspected adult population. In our opinion, the research design used is representative for and exceptionally relevant to clinical practice, primarily because the data were collected during the assessment phase, before the diagnosis had been established.

With regard to possible limitations of the current study, five points should be mentioned. First, there was no non-ASD comparison group without any other diagnoses. In principle this is not necessary, given that our study concerns the use of questionnaires within a clinical setting. It would nevertheless have been interesting to be able to compare the non-ASD but clinically referred group to a matched non-ASD group without any other diagnoses. The AQ and SRS-A scores of our non-ASD group were lower in comparison to the ASD group, but higher in comparison to the general population as reported in the core publications. A similar result was found by Sizoo et al. (2015), using other ASD questionnaires.

Second, it should be noted that 37% of the initial data were omitted due to exclusion criteria. The main reasons for exclusion were inherent to clinical practice (incomplete data and non-compliance to protocol) and impossible to prevent. Nevertheless, the reader has to keep in mind that this does have an effect on the specific group represented here.

Third, raw scores were used in the calculation of optimal cut-offs, while in clinical practice standard scores are preferred. However, the use of raw scores is in accordance with recommended practice for research. This also facilitates the comparability with former studies.

Fourth, the characteristics of the samples could have caused limitations. Group sizes were not equal. The non-ASD group was smaller than the ASD group. Although this is considered statistically suboptimal, this ratio is representative of the population encountered in clinical practice. This ratio is also consistent with results as reported by Sizoo et al. (2015). They found a similar ASD rate (66%) among patients referred to six different outpatient mental healthcare facilities in the Netherlands for purposes of ASD assessment. Furthermore, there was a wide age range, with also older patients included. The age of the ASD group ranged from 19 to 62 years. This is similar to other studies on sensitivity and specificity of AQ and SRS-A (e.g. Ashwood et al. 2016; Baron-Cohen et al. 2001; Sizoo et al. 2015; South et al. 2017). We had only three people of 55+ ; their AQ and SRS-A scores were no outliers or in any other way different from the others. It is not uncommon for people being assessed for the first time in adulthood, even in old age (O’Regan and Tobiansky 2014). For, individuals with ASD born before 1980 may have gone undiagnosed or misdiagnosed or may have had no access to any formal diagnostic process at all (Brugha et al. 2011; Stagg and Belcher 2019). Besides that, it should be mentioned that clinical impression and highest level of education completed are weak indicators of overall level of intellectual functioning. In doubtful cases, IQ testing was performed. However, we cannot claim with certainty that all IQ’s were above 70, since not everyone was tested. This may have had an effect on the results. Also, the presence of co-morbidity in the ASD patients (30%, particularly anxiety and depression) could have had an influence on the results. For instance, Ashwood et al. (2016) found that the AQ seemed more sensitive to the presence of general anxiety disorder (GAD) than it was to ASD. Also, South et al. (2017) found similar SRS-A scores for both people with ASD versus social anxiety. Notwithstanding, extra analyses (not presented here) disregarding patients with co-morbidity did not change our conclusions.

A final, fifth limitation concerns the use of self-report instruments, which depend on self-insight. This can be limited in adults with ASD and could therefore affect the reliability of self-report questionnaires (Baron-Cohen et al. 2001; Hoekstra et al. 2008). However, this study does not focus on the accuracy of self-report questionnaires to reflect aspects of behavior, but on to which extent results of self-report questionnaires are predictive of the ultimate diagnosis. Also, Ashwood et al. (2016) found no differences in predictive value of the AQ between self- and informant report. Although, one can hypothesize that problems with self-report may lead to a higher rate of missed patients. This would be more visible in research where the ASD prevalence rate is higher, as was the case here. When looking at the group of missed patients in our study, the sex ratio is noteworthy. Considerably more men were present (AQ: 80%, SRS-A: 75%, more than the overall sex ratio in our total ASD group: 57% men). Possibly men have more difficulty reflecting and reporting on their own ASD symptoms, than their female counterparts. As a consequence, they would more easily end up as a missed patient.

### Conclusion

Within our general mental healthcare setting, in which ASD patients are mostly seen with a DSM severity level of 1, we conclude that the AQ has a slightly greater predictive value as part of ASD assessment than does the SRS-A. However, we also argue that psychometric data, including predictive values, are neither fixed nor generally applicable. In clinical practice, predictive values have been shown to be poorer than is the case in general psychometric research, as reported in professional manuals and core publications. Although this does not make professional manuals and core publications less relevant, it does highlight the importance of properly considering whether the data are representative of the specific setting. Predictive values are population and setting-specific, and they do not constitute a general characteristic of the actual questionnaire. The values apply only to a well-specified population under well-specified conditions. Self-report questionnaires are widely used within clinical practice. Future research should continue to assess the integration of self-report and clinician-based measures, given that both are associated with strengths and limitations and provide unique information for the assessment process.
